# Oligodendrocyte Birth and Death following Traumatic Brain Injury in Adult Mice

**DOI:** 10.1371/journal.pone.0121541

**Published:** 2015-03-23

**Authors:** Krista A. Dent, Kimberly J. Christie, Nicole Bye, Harleen S. Basrai, Alisa Turbic, Mark Habgood, Holly S. Cate, Ann M. Turnley

**Affiliations:** 1 Department of Anatomy and Neuroscience, The University of Melbourne, Parkville, Australia; 2 Department of Pharmacology and Therapeutics, The University of Melbourne, Parkville, Australia; Hospital Nacional de Parapléjicos—SESCAM, SPAIN

## Abstract

Oligodendrocytes are responsible for producing and maintaining myelin throughout the CNS. One of the pathological features observed following traumatic brain injury (TBI) is the progressive demyelination and degeneration of axons within white matter tracts. While the effect of TBI on axonal health has been well documented, there is limited information regarding the response of oligodendrocytes within these areas. The aim of this study was to characterize the response of both mature oligodendrocytes and immature proliferative oligodendrocyte lineage cells across a 3 month timecourse following TBI. A computer-controlled cortical impact model was used to produce a focal lesion in the left motor cortex of adult mice. Immunohistochemical analyses were performed at 48 hours, 7 days, 2 weeks, 5 weeks and 3 months following injury to assess the prevalence of mature CC-1^+^ oligodendrocyte cell death, immature Olig2^+^ cell proliferation and longer term survival in the corpus callosum and external capsule. Decreased CC-1 immunoreactivity was observed in white matter adjacent to the site of injury from 2 days to 2 weeks post TBI, with ongoing mature oligodendrocyte apoptosis after this time. Conversely, proliferation of Olig2^+^ cells was observed as early as 48 hours post TBI and significant numbers of these cells and their progeny survived and remained in the external capsule within the injured hemisphere until at least 3 months post injury. These findings demonstrate that immature oligodendrocyte lineage cells respond to TBI by replacing oligodendrocytes lost due to damage and that this process occurs for months after injury.

## Introduction

Traumatic brain injury (TBI) is a major cause of death, disability and mental illness on a global scale. Many of the late-onset pathological features of TBI are directly related to aberrant axonal functioning, and result in the progressive atrophy of white matter tracts throughout the brain [1–3]. Injury induced white matter degeneration has been characterized in conjunction with persistent inflammation [4], myelin breakdown [5], and the axonal accumulation of amyloid-β, caspase-3 and other cellular products [6–8].

Oligodendrocytes play a major role in maintaining axonal health in the adult CNS. However, these cells are extremely vulnerable to damage under pathological conditions [9]. There are several reasons for this; firstly oligodendrocytes are susceptible to oxidative damage and function at what is estimated to be the highest metabolic rate of any cell type in the brain [10]. This high energy demand is required for the production and maintenance of large amounts of myelin, yet it also results in the rapid production of toxic metabolites and reactive oxygen species. Oligodendrocytes have a limited capacity to cope with oxidative stress, as they only produce small amounts of the antioxidant, glutathione [11]. Therefore any condition which induces metabolic or oxidative stress is likely to overload these cells and result in apoptosis [12]. The presence of inflammatory cytokines is also known to initiate oligodendrocyte apoptosis. For example, interferon gamma (IFNγ) can cause the death of proliferative oligodendrocyte precursor cells, and tumor necrosis factor α (TNFα) can initiate apoptosis in mature oligodendrocytes [13,14]. Finally oligodendrocytes are susceptible to death through excitotoxicity from the uncontrolled release of glutamate and ATP. This phenomenon is seen in multiple disease states, and causes an increase in oligodendrocyte membrane permeability to extracellular Ca^2+^ influx, resulting in apoptosis [15,16]. All of the aforementioned conditions are features of TBI, i.e. cellular excitotoxicity [17], oxidative stress [18,19], and the release of inflammatory cytokines [20,21]. Since these factors are known to affect oligodendrocytes, it is expected that TBI has an influence on these cells.

Damage to the CNS is also known to activate several cell types which may influence the pathology of oligodendrocytes. Following injury to the brain, among the first cells to enter the site of damage are blood-borne macrophages, along with endogenous microglia [22]. These immune cells accumulate at the site of lesion within hours of injury. Microglia and macrophages of the CNS phagocytose cellular debris and foreign bodies and take part in mediating inflammation, promoting and directing tissue repair, and maintaining cellular homeostasis. Yet, while these cells are essential for the repair and maintenance of the CNS, activity of microglia can also have deleterious effects on local populations of oligodendrocytes and neurons. In times of CNS dysfunction, microglia can release various cytotoxic and pro-inflammatory substances which are known to cause demyelination [23]. Furthermore, evidence has recently emerged that activated microglia can remain in the white matter tracts of TBI patients for up to 18 years following injury. These cells may be involved in long term neuroinflammation that may drive the decay of white matter tracts; possibly through the death of oligodendrocytes [4]. Astrocytes also become activated in the days following TBI [20,24–26]. Astrocytes play a major role in maintaining the condition of both neurons and oligodendrocytes. In the healthy CNS they work to maintain extracellular ion concentrations, prevent excitotoxicity through the uptake of excess glutamate, and minimize oxidative stress through the production of the antioxidant, glutathione [27]. Following CNS injury, the process of glial scar formation acts to rapidly re-establish barrier function, and prevent further tissue damage [22]. However, while this process provides a quick solution to the immediate consequences of CNS injury, astrocytes which make up the glial scar ultimately prevent long term recovery. They secrete factors that inhibit the regrowth of axons, which in turn results in the death of oligodendrocytes. However, these cells may also prevent white matter repair and remyelination, as reactive astrocytes can impede the migration of oligodendrocyte precursor cells [28]. Overall, it still remains unclear how the presence of activated cells such as microglia and astrocytes may influence oligodendrocyte pathology following TBI.

Current lines of evidence support the concept that oligodendrocyte populations undergo a dynamic range of responses during the acute phase of brain injury. White matter oligodendrocyte numbers decline within the first week following fluid percussion brain injury in the rat [29] and a rapid increase in the number of oligodendrocyte progenitors throughout both grey and white matter regions proximal to the site of injury have been reported [5,30–33]. There is some evidence that microglia, macrophages and reactive astrocytes may be involved in driving the proliferation and recruitment of oligodendrocyte precursor cells following CNS injury [22,34]. While the short term influences of TBI on oligodendrocyte populations are becoming apparent, little is known about how these cells respond in the months following injury.

The purpose of this study was to investigate the dynamics of oligodendrocyte populations within the corpus callosum and external capsule for up to 3 months following unilateral cortical impact injury in the mouse. A computer-controlled cortical impact model of TBI was used [35]. Immunohistochemical assays were performed at selected timepoints ranging from 48 hours to 3 months post TBI. Populations of oligodendrocytes were monitored for changes in total cell number across time and the extent of apoptotic cell death was investigated by analysis of activated caspase-3. Since previous studies have documented an increase in oligodendrocyte precursor cell numbers in response to TBI, EdU was administered to assess the proliferation and survival of newly formed oligodendrocytes and their CC-1^+^ progeny.

Since the health of axons relies on the efficient functioning of oligodendrocytes, investigating how this cell type responds to TBI may be an important factor in understanding the mechanisms of white matter degeneration over time. The results of this study demonstrate that early oligodendrocyte lineage proliferation and ongoing mature oligodendrocyte apoptosis have long lasting effects on the pathology of white matter oligodendrocytes for months following TBI.

## Materials and Methods

### Animals

Adult C57Bl/6 mice of mixed gender (aged 7–11 weeks, *n =* 40 total; *n =* 25 female, *n =* 15 male) were purchased from the Animal Resource Centre (ARC; Perth, Australia). Animals were housed in a SPF facility, on a 12:12 light/dark cycle and allowed to acclimatize for at least 1 week prior to surgery. After injury animals were single housed in individually ventilated cages and allowed free access to food and water. The experimental use of animals for this project was approved by the University of Melbourne’s Animal Ethics Committee (Approval number: 11–083) and all procedures followed current regulations set by the National Health and Medical Research Council, Australia.

### Surgery—Controlled Cortical Impact Model of TBI

Mice were randomly assigned to TBI or sham injury groups. Five survival time-points were established post TBI, with animals killed at 48 hours, 7 days, 2 weeks, 5 weeks and 3 months following injury (*n =* 4 sham, *n =* 4 TBI except for the 3 month sham group with n = 3). There were no mortalities in the TBI group but one sham injured mouse died immediately following surgery. All other animals remained healthy and gained weight over the course of the study.

Animals were anaesthetized with 5% Isoflurane and administered meloxicam (1mg/kg; Troy-Ilium, Glendenning, NSW, Australia) for analgesia. The animal was positioned on a stereotaxic frame (Kopf Instruments, CA, USA) while 2% Isoflurane continued to be supplied via the nose-cone. The cranium was exposed via an incision along the midline of the scalp. Using a dentist drill, a 3mm unilateral (left) craniotomy was performed at stereotaxic coordinates 2.5mm lateral to Bregma. The computer-controlled cortical impactor design was essentially as per previously used [36]. The impactor tip (1.8mm diameter) was positioned at the surface of the brain and was programmed to produce a 2mm deep impact with a dwell time of 1 second. This injury produces a focal lesion affecting the left motor cortex and some somatosensory cortex.

Following injury, a small plastic cap was fixed over the hole in the skull with Loctite 454 gel and the scalp was sutured back together. Sham injured animals received the same treatment as injured mice, except the impact procedure was omitted. Mice regained consciousness and became active within 5–10 minutes of surgery and were monitored from the day of surgery up until 6 days post injury and then once weekly thereafter.

### Administration of EdU

To follow the proliferation of cells in response to TBI, all animals were administered 50mg/kg EdU (5-ethynyl-2’-deoxyuridine; Invitrogen-Molecular Probes, Eugene, OR, USA) via intraperitoneal injection once daily from the day of injury, up until 6 days post injury. The EdU stock concentration was 10mg/ml.

### Tissue preparation

Animals were administered a lethal dose of 50% Pentobarbitone (Lethabarb, Virbac, Milperra, VIC, Australia). Once anaesthetized, mice were perfused transcardially with 20mL 0.1M phosphate buffered saline (PBS) followed by 20mL 10% neutral buffered formalin (NBF) (Grale Scientific, VIC, Australia). Brains were collected and post-fixed by immersion in 10% NBF for 30 minutes on ice, before being stored in 30% sucrose in 0.1M PBS at 4°C for up to 2 days after collection. The tissue was then placed in Tissue-Tek O.C.T. (Sakura, The Netherlands) before being snap frozen in isopentane. Coronal sections (10μm) were cut on a cryostat (Leica, Nussloch, Germany) and mounted directly onto slides where they were allowed to dry before storage at −80°C.

### Immunohistochemical staining

The slide mounted sections were defrosted via immersion in 0.1M PBS for 15 minutes. Non-specific binding was blocked with 5% normal goat serum + 0.1% triton in PBS for 1 hour. Primary antibodies were diluted in blocking solution: mouse anti-CC-1 (OP80-100UG; Calbiochem, CA), rabbit anti-Olig2 (AB9610; Chemicon, CA), mouse anti-CNPase (MAB1580, Millipore, Australia) and rabbit anti-cleaved caspase-3 (#9661; Cell Signaling Technologies, MA) and incubated on sections overnight at room temperature. After washing, secondary antibodies Alexa Fluor-488 (anti-mouse, anti-rat or anti-rabbit; Invitrogen, OR, USA), or Cy-3 (anti-rabbit; Invitrogen, OR, USA), were diluted in PBS and incubated for 1.5 hours at room temperature. Sections were then washed 3 times in PBS+DAPI before being coverslipped with fluorescent mounting medium (Dako, Glostrup, Denmark).

### EdU detection

The above protocol was followed except that primary antibody incubation time was reduced to 2hours. Following the secondary antibody, the sections were washed with PBS and then blocked with 5% bovine serum albumin (BSA) + 0.5% Triton X100 in PBS for 30 minutes. EdU staining was then performed using the ClickIt-Alexa Fluor-555 EdU imaging kit (Invitrogen).

### Cell counts

Images were captured using an Olympus IX81 inverted fluorescent microscope with camera. All images to undergo counts were taken at x20 magnification with uniform camera settings. Defined regions of interest were selected for quantification of cell numbers in the corpus callosum and the lateral aspect of the external capsule and the area of each region per section was measured and counts converted to number per mm^2^. Counts were performed in both the ipsilateral (injured) and contralateral hemispheres of the brain. Cell counts performed manually using ImageJ software (Wayne Rasband, NIH http://imagej.nih.gove/ij). Sections at −0.7mm, 0.0mm and 0.7mm relative to Bregma were used for analysis (n = 3 sections/mouse). The mean cell count for each mouse was used to generate a group mean from 3–4 mice at each timepoint.

### Statistical analysis

One-way ANOVA and associated Bonferroni post-hoc tests were performed using GraphPad Prism 5. Statistical significance was assumed for *P* values of ≤0.05. All graphs are shown as the mean of group data with error bars representing the standard error of the mean (±SEM).

## Results

### Location of TBI injury and regions analyzed

**Fig 1:**
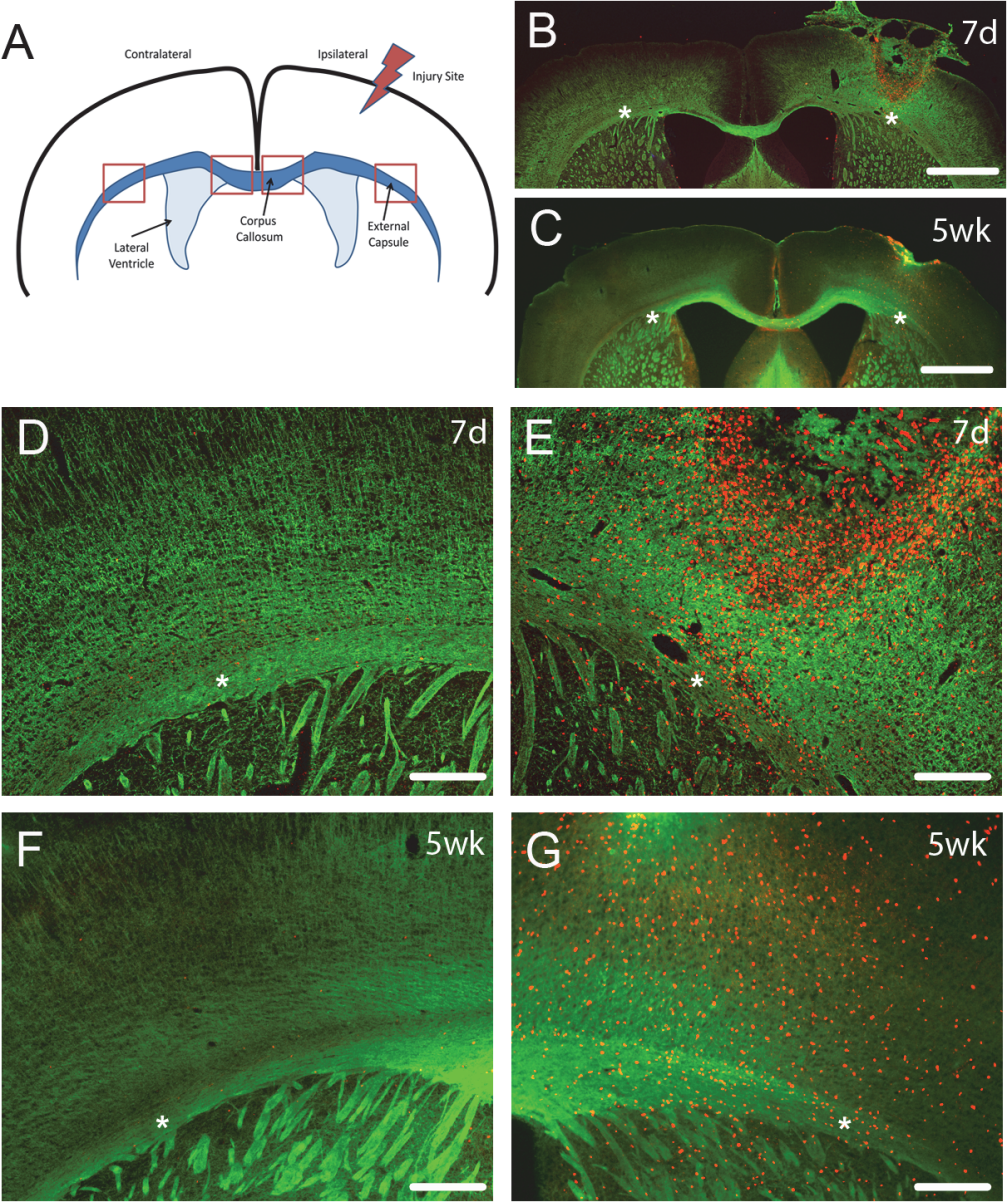
Diagram of TBI location and CNPase staining of myelin fibers. (A) The location of the TBI was in the cortex above the external capsule, with analyses performed on oligodendrocyte lineage cells in the ipsilateral and contralateral external capsule and corpus callosum (regions analyzed indicated by boxes). CNPase staining (B-D) of myelin (green) and EdU (red) labelling of proliferative cells at 7 days (7d) and 5 weeks (5wk) after TBI showed that this injury largely affected the cortex but also perturbed myelin tracts in the ipsilateral external capsule (B, C, E, G) with few EdU labeled cells on the contralateral side (D, F). This did not usually result in extensive long term myelinated fiber loss, as indicated by CNPase staining at 5 weeks (wk) after TBI, with ipsilateral external capsule showing similar levels of myelination as contralateral external capsule. Scale bars in B,C 1mm; D-G 200μm. Asterisks in B-G indicate the external capsule with panels D and E an enlargement of B and panels F and G an enlargement of C.

The controlled cortical impact model we used primarily injured cortex, and variably directly affected underlying myelinated fibers of the external capsule. The corpus callosum was not directly injured in this model (Fig. 1). CNPase immunostaining for myelin shows that there was some disruption of myelin in ipsilateral external capsule at 7d post TBI but return of myelin staining at 5 weeks after TBI (Fig. 1 B-G).

### TBI results in a loss of CC-1 immunoreactivity

**Fig 2:**
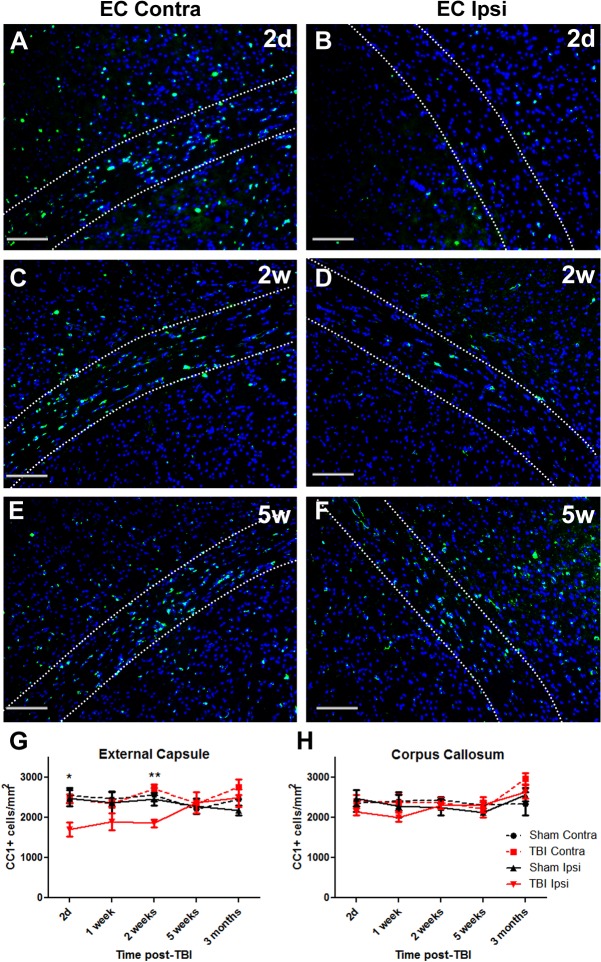
CC-1^+^ immunostained cell numbers decrease by 2 days after TBI and return to control levels by 5 weeks. Brain sections from TBI mice were immunostained for mature CC-1 expressing oligodendrocytes and counterstained with DAPI. Panels show contralateral (Contra) and ipsilateral (Ipsi) external capsule (EC) at (A,B) 2 days (2d), (C,D) 2 weeks (2w) and (E,F) 5 weeks (5w) (G) In the external capsule numbers of CC-1^+^ oligodendrocytes were decreased by 2d after injury, remained decreased at 2 weeks and returned to normal levels by 5 weeks. (H) There was no significant loss of CC-1^+^ oligodendrocytes in the corpus callosum. Results in panels G and H show mean+/-SEM of sections from n = 3–4 mice per timepoint. Statistical comparisons were made using ANOVA (F_(19,58)_ = 2.281, *p*<0.01 for external capsule in G, F_(19,54)_ = 1.51, *p* = 0.12 for corpus callosum in H) and comparisons were made between ipsilateral TBI and sham at each timepoint using the Bonferroni *post hoc* test; *p<0.05, **p<0.01.

The CC-1 antibody was used to label populations of mature oligodendrocytes. Numbers of CC-1/DAPI positive cells were counted within the corpus callosum and external capsule in both ipsilateral and contralateral hemispheres of the brain. Fig. 2A–F show CC-1 staining within the contralateral and ipsilateral external capsule at 2 days, 2 weeks and 5 weeks post TBI.

There were no statistically significant differences in cell counts in sham-injured regions of interest across time. CC-1 positive cell numbers were compared between contralateral and ipsilateral sides of the brain of TBI mice. By 2 days post TBI, numbers of CC-1 immunoreactive cells within the ipsilateral external capsule were significantly decreased compared to sham sections and remained significantly decreased at the 2 week time point (Fig. 2G). There were no significant differences in CC-1^+^ cell numbers in the corpus callosum over this time period (Fig. 2H).

### TBI induces caspase-3 mediated CC-1^+^ cell apoptosis

**Fig 3:**
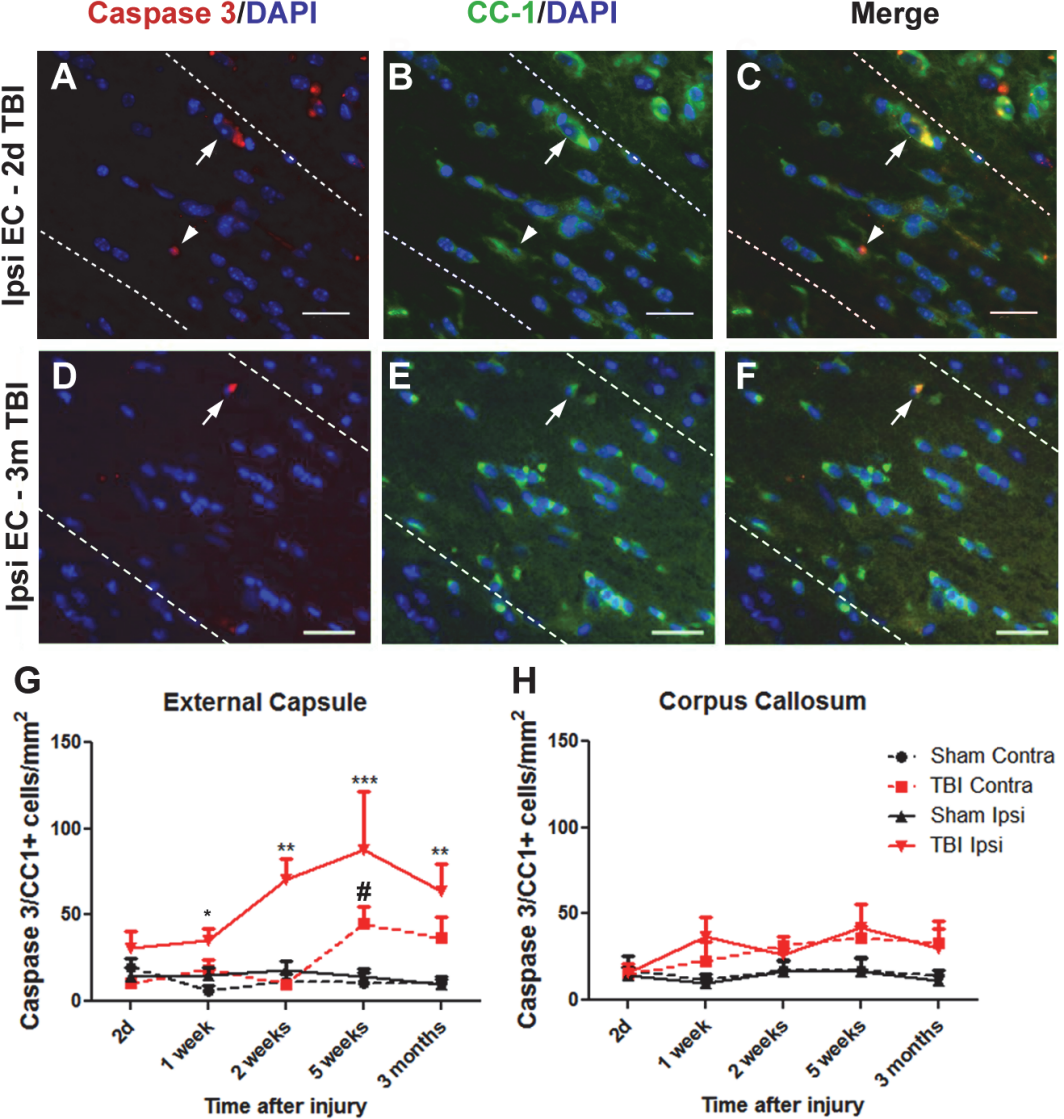
Activated Caspase-3^+^/CC-1^+^ cell numbers increase to a maximum at 5 weeks after TBI. Brain sections were immunostained for activated caspase-3 and CC-1 and counterstained with DAPI. Panels show ipsilateral external capsule (EC) of brains at 2d (A-C) and 3 months (D-F) post-TBI: (A, D) Caspase-3 and DAPI, (B, E) CC-1 and DAPI, (C F) merged. Arrows show co-labelled cells, indicative of apoptotic oligodendrocytes. In panels A-C, (G) the arrows indicate an early apoptotic CC-1^+^ oligodendrocyte with cytoplasmic CC-1 and Caspase-3 staining and a pyknotic nucleus, while the arrowhead indicates a later apoptotic cell where Caspase-3 is present in the nucleus. In the ipsilateral external capsule, numbers of Caspase-3^+^/CC-1^+^ oligodendrocytes were significantly increased by 1 week after injury. Apoptotic oligodendrocytes were also elevated in the contralateral external capsule by 5 weeks. (H) No significant oligodendrocyte apoptosis was detected in the corpus callosum. Results in panels D and E show mean+/-SEM of sections from n = 3–4 mice per timepoint. Statistical comparisons were made using ANOVA (F_(19,58)_ = 4.637, *p*<0.0001 for external capsule in D, F_(3,12)_ = 5.638, *p* = 0.01 for the 1 week timepoint in D and F_(19,58)_ = 1.569, *p* = 0.097 for corpus callosum in E) and comparisons were made between ipsilateral TBI and sham at each timepoint using the Bonferroni *post hoc* test; *p<0.05; **p<0.01, **p<0.001; # Contralateral TBI was significantly different to sham by t-test, *p*<0.05.

Caspase-3 is a key mediator of apoptosis. CC-1 and activated caspase-3 double immunostaining was used to evaluate the extent of apoptotic cell death within mature oligodendrocyte populations. The majority of caspase-3 labelled cells were seen around the injury site, but a few cells were observed throughout the parenchyma of sham injured tissue as well as within the contralateral hemisphere of TBI brains, many of which did not co-label with CC-1 (data not shown).The majority of caspase-3/CC-1^+^ cells were identified in ipsilateral external capsule at all timepoints as shown for 2 days (Fig. 3A–C) and 3 months (Fig. 3D–F) post-TBI.

Caspase-3^+^/CC-1^+^/DAPI^+^ co-labelled cells were counted in corpus callosum and external capsule as above. At 48 hours post injury there was a non-significant trend towards increased caspase-3^+^/CC-1^+^ cells within the ipsilateral external capsule, which increased at 7 days post injury and remained increased to 3 months (Fig. 3G). The corpus callosum did not show any significant change in CC-1 apoptotic cell death (Fig. 3H).

### Olig2^+^ cells are stimulated to proliferate following brain trauma and are capable of long term survival

**Fig 4:**
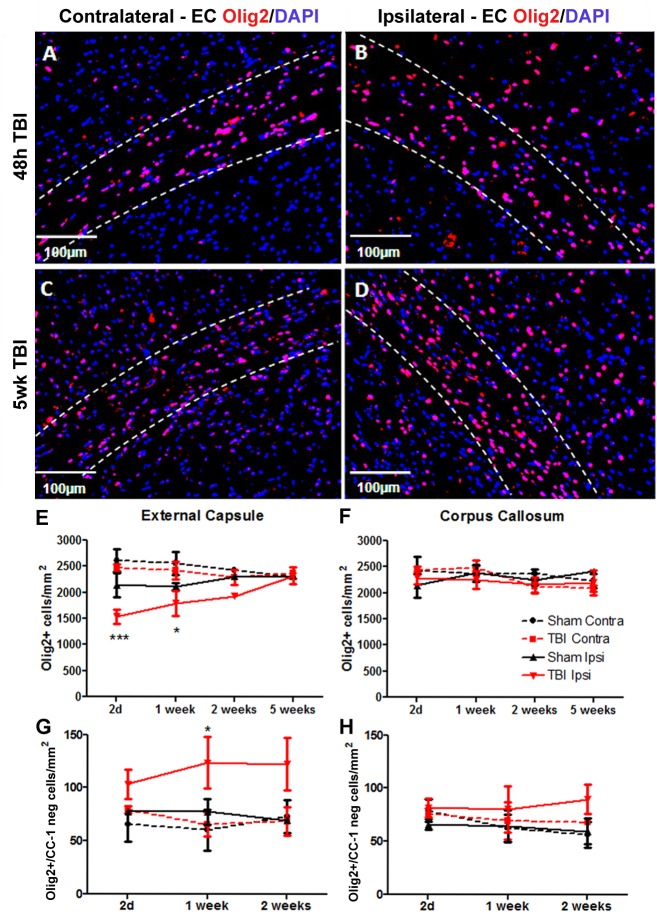
Olig2 cells in external capsule at 48h and 5 weeks after TBI. Brain sections were immunostained for Olig2 and counterstained with DAPI. Panels show (A,C) contralateral and (B,D) ipsilateral external capsule (EC) at (A,B) 48h and (C,D) 5 weeks (5wk) post-TBI. The bordered regions indicate external capsule. (E) In the external capsule numbers of Olig2^+^ oligodendrocytes were decreased by 2d after injury, remained decreased at 1 week and returned to normal levels by 2 weeks. (F) There was no significant loss of Olig2^+^ oligodendrocytes in the corpus callosum. (G) There was an increase in numbers of Olig2^+^/CC-1 negative cells at 1 week post-injury but (H) no increase in these cells in the corpus callosum. Results in panels E-H show mean+/-SEM of sections from n = 3–4 mice per timepoint. Statistical comparisons were made using ANOVA (F_(15,38)_ = 3.624, *p*<0.001 for external capsule in E, F_(11,30)_ = 2.062, *p* = 0.05 for external capsule in G) and comparisons were made between ipsilateral TBI and sham at each timepoint using the Bonferroni *post hoc* test; *p<0.05, ***p<0.001.

Olig2 is a transcription factor that is expressed in both mature oligodendrocytes as well as oligodendrocyte precursor cells [37] and was used here as a general marker of oligodendrocyte lineage cells. Fig. 4 shows the staining pattern of Olig2-labeled cells at 48 hours and 5 weeks post injury within the contralateral and ipsilateral external capsule. There was a decrease in Olig2^+^ cell numbers in ipsilateral external capsule at 48hrs and 1 week post-TBI with numbers returning to control levels by 2 weeks (Fig. 4E). This was mirrored by an increase in the number of Olig2^+^/CC-1 negative cells (immature oligodendrocyte lineage cells) (Fig. 4G). There was no effect of TBI on total Olig2^+^ cell numbers or Olig2^+^/CC-1 negative cell numbers in corpus callosum (Fig. 4F, 4H).

**Fig 5:**
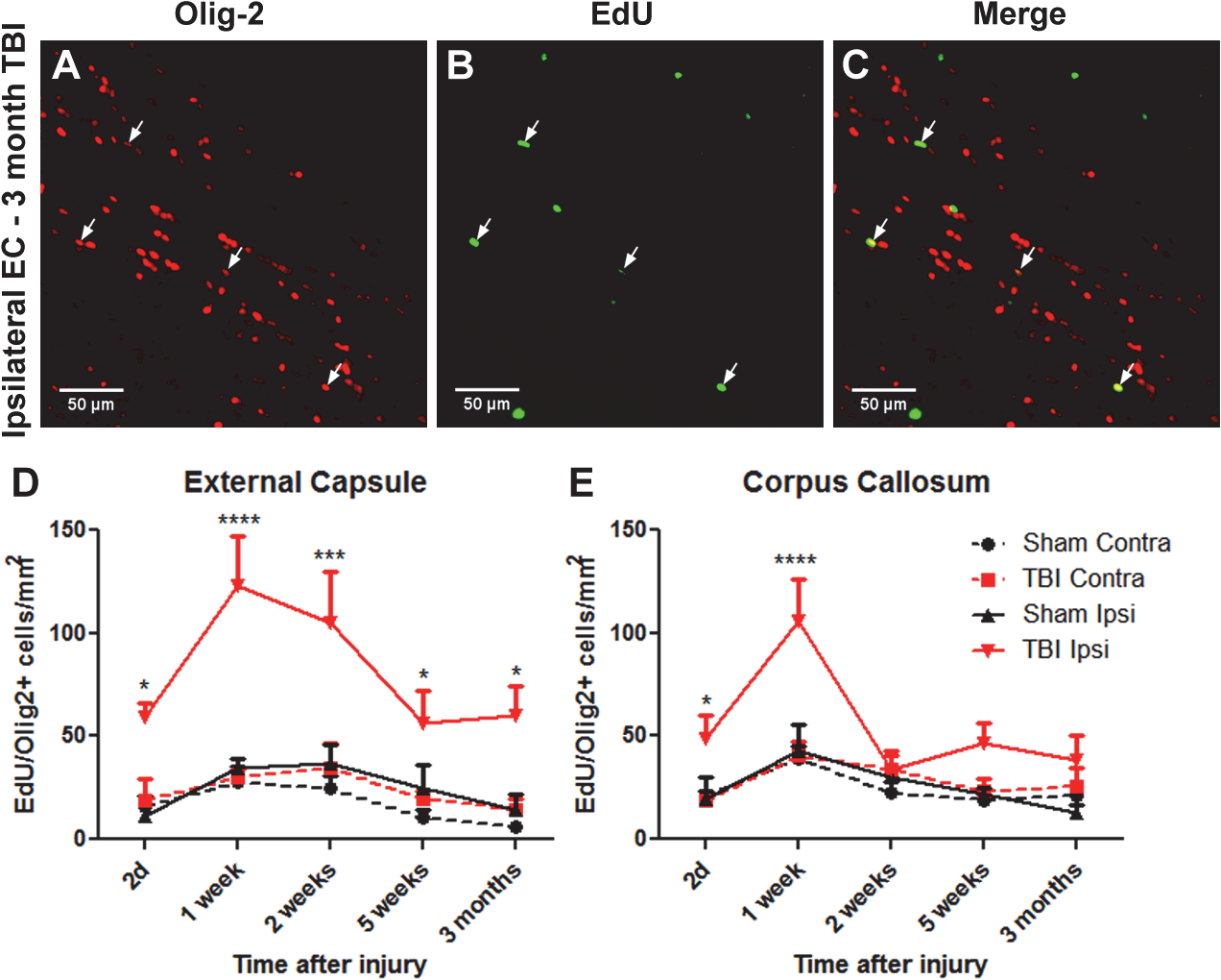
Proliferative Olig2 cell numbers increase within 2d after TBI and remain elevated to at least 3 months. Brain sections were immunostained for Olig2 and counterstained for EdU and DAPI. Panels show ipsilateral external capsule (EC) of a 3 month post-TBI brain: (A) Olig2, (B) EdU, (C) merged. Arrows show some co-labelled cells, indicative of oligodendrocytes generated after TBI. (D) In the ipsilateral external capsule, numbers of EdU^+^/Olig2^+^ oligodendrocytes were significantly increased within 2d after injury, were further increased at 1 and 2 weeks, with lower numbers by 3 months. (E) A significant increase in EdU^+^ oligodendrocytes was also detected in the corpus callosum between 2d and 1 week, decreasing to control levels by 2 weeks. Results in panels D and E show mean+/-SEM of sections from n = 3–4 mice per timepoint. Statistical comparisons were made using ANOVA (F_(19,58)_ = 7.545, *p*<0.0001 for external capsule in D and F_(19,58)_ = 4.808, *p*<0.0001 for corpus callosum in E) and comparisons were made between TBI and sham at each timepoint using the Bonferroni *post hoc* test; *p<0.05, ***p<0.001.

Co-labeling of Olig2 and EdU was then assessed (Fig. 5A–C). Double labeled Olig2^+^/EdU^+^ cells represent a mixed population of newly formed oligodendrocyte precursor cells and, at later timepoints, mature oligodendrocytes. EdU labelled cells were observed throughout the ipsilateral hemisphere of injured brains. In the contralateral hemisphere EdU^+^ cells were mainly limited to white matter regions. Within 48 hours of injury, the number of Olig2^+^/EdU^+^ cells within the ipsilateral external capsule and corpus callosum had increased to levels approximately four times greater than sham levels (Fig. 5D, 5E). By 7 days, with daily administration of EdU over that period, both corpus callosum and external capsule demonstrated greater Olig2^+^/EdU^+^ cell numbers. By 2 weeks Olig2^+^/EdU^+^ cell counts declined, however their numbers remained elevated compared with shams, until at least 3 months in the ipsilateral external capsule but not corpus callosum.

### EdU labelled mature CC-1^+^ cells after TBI

**Fig 6:**
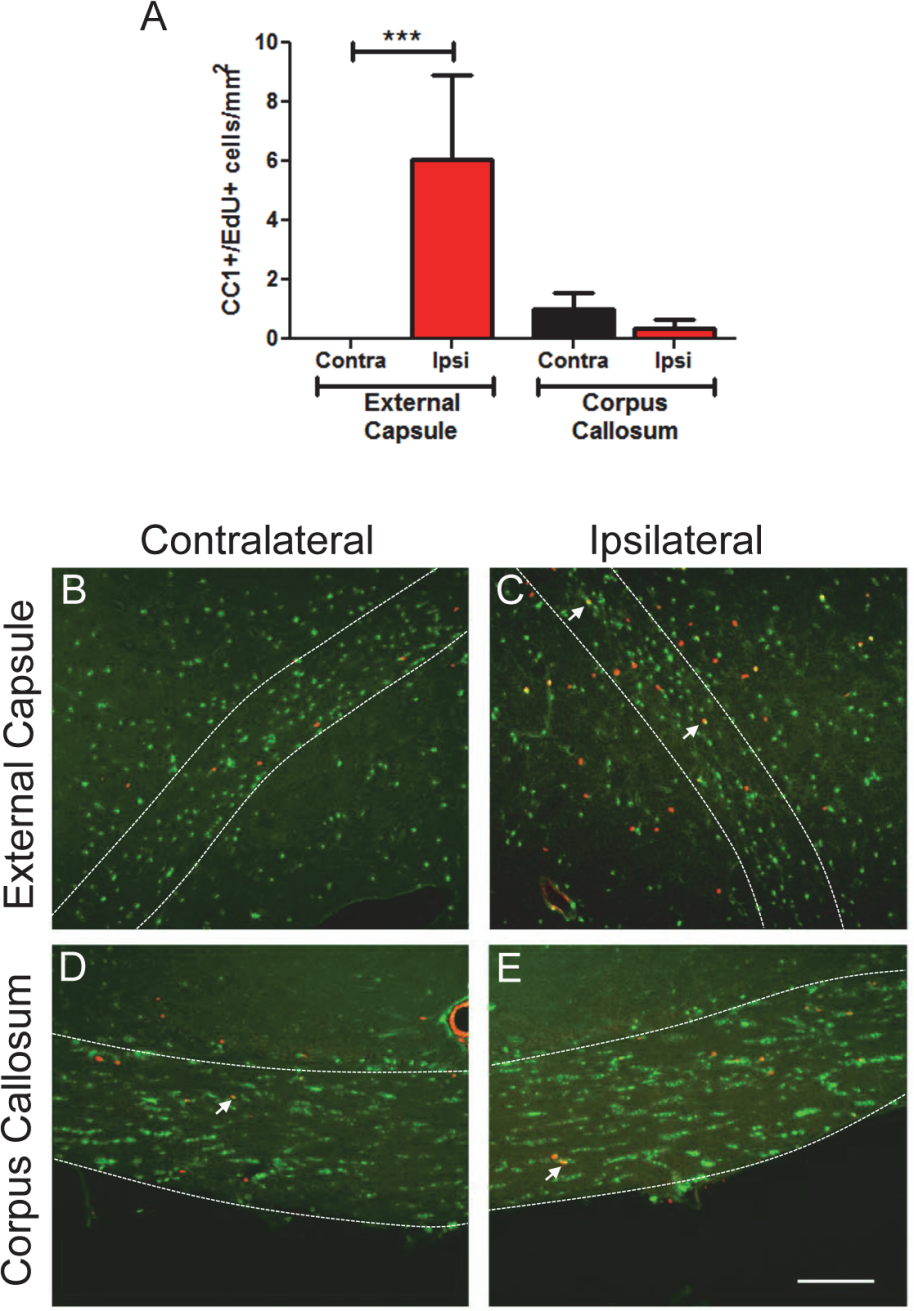
EdU^+^/CC-1^+^ cells are present to at least 3 months post-TBI. Brain sections were immunostained for CC-1 and counterstained with EdU and DAPI. (A) Increased numbers of EdU^+^/CC-1^+^ cells were present in ipsilateral external capsule at 5 weeks post injury, compared to the contralateral side or corpus callosum. EdU^+^/CC-1^+^ cells were also present at 3 months post-TBI. Panels show (B,C) external capsule and (D,E) corpus callosum at 3 months post-TBI. Examples of EdU^+^/CC-1^+^ oligodendrocytes are indicated by arrows. The bordered regions indicate external capsule or corpus callosum respectively. Scale bar 50μm. Results in panel A show mean+/-SEM of sections from n = 3 mice per group. Statistical comparisons were made using ANOVA (F_(3,8)_ = 3.784, *p* = 0.05) and comparisons were made between ipsilateral and contralateral sides using the Bonferroni *post hoc* test; *p<0.05.

To determine whether the generation of proliferative Olig2 oligodendrocyte precursor cells differentiated into mature oligodendrocytes, sections at 5 weeks and 3 months post TBI were immunostained for CC-1 and EdU. Increased numbers of CC-1^+^/EdU^+^ cells were found in the ipsilateral external capsule at 5 weeks (Fig. 6A). CC-1^+^/Edu^+^ cells were also present in the corpus callosum, however there was no obvious difference between the numbers of double positive cells in the ipsilateral and contralateral corpus callosum. EdU^+^/CC-1^+^ oligodendrocytes remained in external capsule and corpus callosum until at least 3 months post-injury (Fig. 6B–E).

## Discussion

We have demonstrated here that following a controlled cortical impact TBI in adult mice there is ongoing mature oligodendrocyte apoptosis with concomitant generation of newborn oligodendrocytes that differentiate into mature CC-1^+^ oligodendrocytes and remain up to at least 3 months after injury, particularly in the subcortical ipsilateral external capsule directly below the cortical injury site.

### The loss of mature oligodendrocytes

The findings presented demonstrate that cortical-impact injury induces a transient reduction in CC-1 immunoreactivity within external capsule ipsilateral to the site of injury (Fig. 2). Although this decrease in immunoreactivity correlated with co-localization of CC-1 with apoptotic markers and likely represents a true loss of mature oligodendrocytes, other possibilities, such as protein marker down-regulation [38] may also influence cell counts. Reduced expression of oligodendrocyte proteins can occur following injury, including myelin basic protein (MBP) and proteolipid-protein (PLP) [39–41]. Loss of CC-1 immunoreactivity has also been reported following brain injury in the rat [29] and spinal cord injury in mice [42]. This loss of CC-1 labelled cells following spinal cord lesion was determined to be result of oligodendrocyte death rather than marker down-regulation. To determine whether our results showed oligodendrocyte loss or marker downregulation, electron microscopy, which does not rely on maker expression, will be performed in future studies.

We also observed a modest but delayed increase in mature oligodendrocyte apoptosis in the contralateral external capsule at 5 weeks post-injury, although no significant increase was found in the corpus callosum at any timepoint. These structures were not directly damaged but may be affected by secondary events, such as ipsilateral neuron damage/death and subsequent axonal loss on the contralateral side, leading to associated oligodendrocyte death. There was a trend to increased oligodendrocyte apoptosis in corpus callosum, but this was not significant except when ipsilateral and contralateral data were combined for sham and TBI, in which case there was a significant increase at 1 and 5 weeks post-injury compared to sham (data not shown).

Previous studies on CNS injuries have shown that the temporal pattern of oligodendrocyte apoptosis can be quite variable, and is obviously dependent on the type, severity and location of trauma. However, it has been consistently demonstrated that oligodendrocytes can undergo a delayed pattern of death, with the wave of apoptosis first affecting oligodendrocytes closest to the site of injury and spreading into more distal populations thereafter. In relation to TBI, lateral fluid percussion injury models show that apoptosis can be seen in white matter oligodendrocytes from as early as 12 hours post injury within the ipsilateral hemisphere, peaking at around 1-week [38]. Through the assessment of several white matter regions, Flygt *et al*. [5] were able to track the spatiotemporal progression of oligodendrocyte death following lateral fluid percussion injury in the rat. In this study the wave of apoptotic activity was first detected in oligodendrocytes of the ipsilateral external capsule, reaching a peak at 2 days. From here more distal regions were affected, with both the corpus callosum and ipsilateral fimbriae experiencing their maximum rate of apoptosis at 7 days. This is earlier than the wave of apoptosis observed in the current study and may reflect differences in injury model and severity of injury used.

### Replacement of oligodendrocytes through oligodendrocyte precursor cell proliferation

While CC-1^+^ cell numbers were diminished throughout the initial 2-weeks of injury in the ipsilateral external capsule, this was offset by a rise in the number of newborn Olig2^+^ cells within the first weeks of injury. At this stage the majority of new Olig2^+^ cells are likely to represent oligodendrocyte precursor cells rather than mature oligodendrocytes and may explain why CC-1^+^ staining was decreased despite the generation of Olig2^+^ cells, as suggested by the increased numbers of Olig2+/CC-1 negative cells over this time period. In addition, although there was no significant oligodendrocyte loss in the corpus callosum post-injury, there was a transient increase in the number of EdU-labelled Olig2^+^ cells for a week after injury. Compared to the total number of Olig2^+^ cells, the number of newborn cells was low and did not affect total Olig2^+^ counts but nonetheless indicates that factors released in response to injury had a broader effect than at the injury site alone.

Following a demyelinating event, it takes approximately four days for a newly formed oligodendrocyte precursor cell to begin expressing early myelin proteins and up to 2-weeks before late oligodendrocyte differentiation markers, such as myelin oligodendrocyte glycoprotein (MOG), are expressed [43]. Overall, it seems that apoptotic oligodendrocyte death is the primary reason for the post-TBI reduction of CC-1^+^ cells. However, numbers of CC-1^+^ cells remained constant between 5 weeks and 3 months despite the peak levels of caspase3/CC-1^+^ cells at these times. This is likely due to the small number of cells dying compared to the total number of CC-1^+^ cells remaining, along with oligodendrocyte replacement, such that a difference in total cell numbers could not be detected.

The death of oligodendrocytes results in the loss of axonal myelin [44], leading to diminished axonal conduction speeds and hence, neurological functioning [45]. Following a demyelinating injury, the process of remyelination can begin within days [43], however whether this is the case following TBI, where neuronal and axonal damage also occurs, is not clear. Further, surviving oligodendrocytes may be incapable of remyelination [46]. Therefore a rapidly proliferating source of new oligodendrocytes is required to both replace cells lost through apoptosis and promote remyelination throughout sites of damage.

### The proliferation of oligodendrocyte precursor cells

In normal adult CNS, the vast majority of Olig2^+^ cells express the oligodendrocyte precursor cell markers NG2 and PDGFR. These cells remain proliferative and produce newborn mature oligodendrocytes, with ongoing myelin remodeling throughout adulthood in both myelinated white matter tracts and grey matter [47,48]. These oligodendrocyte precursor cells are capable of increased proliferation in times of injury or demyelination [49–51]. The present study shows that following TBI there is an expansion in the number of newly formed oligodendrocytes within ipsilateral white matter regions during the first weeks of injury. A number of other studies have also documented the proliferation of oligodendrocyte precursor cells in this initial phase of injury [5,30,52]. Indeed, the peak in total oligodendrocyte precursor cell numbers appears to occur sometime within the first week of injury, with proximal grey and white matter regions both affected [5,30–33]. In the current study EdU was administered daily throughout the first week of injury, and by the 7 day time point the number of EdU labelled Olig2^+^ cells reached a maximum, supporting previous studies. It is likely that enhanced oligodendrocyte precursor cell proliferation continues past this time point but it is unclear to what extent this occurs; no expansion in EdU^+^ cell numbers was detected after EdU administration was ceased indicating that the newborn Olig2^+^ cells did not continue to proliferate.

### The long term outcome of newly formed oligodendrocytes

Following their peak at 7 days post injury, numbers of newly formed immature oligodendrocytes and their progeny decreased. This decline may be due to either ongoing apoptotic cell loss, or progressive EdU marker de-labelling. One of the issues with EdU labelling is the gradual dilution of the EdU marker after multiple rounds of cell division [53]. This marker loss can occur rapidly if newborn cells undergo a period of post EdU transit amplification before they enter into terminal differentiation. At 3 months post TBI, EdU labelled Olig2^+^ cells could still be seen throughout the ipsilateral external capsule. These remaining cells may represent either a quiescent or slowly dividing population of oligodendrocyte precursor cells, or they may represent fully differentiated oligodendrocytes capable of myelination. Given that many EdU^+^ cells at 5 weeks and 3 months post TBI were also CC-1^+^, it suggests the latter. This observation indicates that transit amplification of EdU^+^ immature oligodendrocytes past the first week of TBI is limited, as newly formed oligodendrocytes could successfully reach maturity while still retaining their EdU content.

Therefore, the progressive loss of EdU labelled oligodendrocytes may result from apoptotic mechanisms. Elevated numbers of apoptotic oligodendrocytes were seen in the ipsilateral EC up until the 3 month experimental endpoint, indicating that apoptosis is likely to be the main contributing factor driving the loss of new oligodendrocytes. Fig. 2 shows that at later time points the number of mature oligodendrocytes in the ipsilateral external capsule returned to control levels rather than declining with the ongoing rate of apoptosis. This suggests that oligodendrocyte precursor cells within the area may be continuing to divide, providing a constant supply of new oligodendrocytes.

The concept that ongoing oligodendrocyte apoptosis, and presumably oligodendrocyte precursor cell proliferation can continue for months following TBI poses the question as to whether this phenomenon is a form of long term remodeling and healing, as has been shown for the normal healthy adult CNS [48] or whether it may be a sign of failing attempts at regeneration. In the field of multiple sclerosis research, it has been hypothesized that ongoing attempts to replace oligodendrocytes and repair demyelinated lesions may result in the eventual depletion of oligodendrocyte precursor cell populations [54]. This has not been characterized in the TBI literature. However, if oligodendrocyte death continues at an accelerated rate in damaged white matter tracts, as shown in the current project, it is possible that local populations of oligodendrocyte precursor cells may also become depleted with time. This may be an important concept to consider in regards to the long term white matter pathology observed in TBI patients.

## Conclusions

As a whole, the findings presented in this project highlight the long term effects of cortical injury at many functional levels. A substantial loss of mature oligodendrocytes within the external capsule was observed within the first 2 weeks following injury, with numbers returning to normal by 5 weeks. Populations of oligodendrocyte lineage cells were shown to undergo significant proliferative activity within the first week of injury and longer term tracking revealed that these cells, along with their differentiated progeny are capable of surviving for months following TBI. This evidence suggests that the successful replacement of lost oligodendrocytes may be possible. Yet, while long term cell survival was possible, there was also evidence of ongoing oligodendrocyte apoptosis, which may prevent overall recovery. Taken together, these findings indicate that white matter oligodendrocyte pathology is a feature of both the acute and chronic phases of TBI. Defining the cause of ongoing oligodendrocyte death will be an important factor in designing therapeutic strategies and preventing the breakdown of white matter regions.
